# Supplementary material for: A closer look at cross-validation for assessing the accuracy of gene regulatory networks and models
Source: Sci Rep. 2018 Apr 26;8:6620. doi: 10.1038/s41598-018-24937-4 (PMC5920056; doi:10.1038/s41598-018-24937-4)
Supplement: Supplementary file 1 — Supplementary Material [file 41598_2018_24937_MOESM1_ESM.pdf]

Supplementary Material for "A closer look at cross-validation for assessing the accuracy of gene regulatory networks and models"

Shayan Tabe-Bordbar, Amin Emad, Sihai Dave Zhao, Saurabh Sinha

SUPPLEMENTARY RESULTS:

Comparison of random and clustered cross-validation using a biased dataset

We assessed the relative behavior of RCV and CCV evaluation procedures on a dataset that does not have equal representation of experimental conditions. We selected 864 patient samples from the PanCan12 dataset such that the number of samples of each cancer type is proportional to the number of samples in the original PanCan12 data. These 864 samples are henceforth called the biased dataset. More specifically, we used 41 samples from LAML, 29 from BLCA, 202 from BRCA, 45 from COAD, 39 from GBM, 72 from HNSC, 114 from KIRC, 84 from LUAD, 62 from LUSC, 63 from OV, 24 from READ, and 89 from UCEC.

The gene expression prediction problem for this dataset was described in Section 'Overview of the gene expression prediction problem and cross-validation strategy', as the task of predicting a gene's expression in a sample based on the expression of 1239 TFs in that sample. For this test, we selected 100 target genes, which had the highest expression variance across the 864 samples in the biased set. We used Elastic Net to perform the gene expression prediction task, since it had the best average performance in most of our previous tests. Supplementary Fig. S2 shows the performance of the prediction algorithm for the 100 examined genes, as quantified by Pearson correlation coefficient for 20 constructed partition collections. Distinctness of each cross-validation partition collection was computed as described in Section 'A new measure to quantify the distinctness of training and test sets' and is shown in Supplementary Fig. 3A. Analysis of the results was performed as described in Sections 'Random and clustered cross-validation produce different estimates of error' and 'Gene expression prediction accuracy estimated by cross-validation is highly correlated with distinctness score of test conditions' of the manuscript. As an example, Supplementary Fig. 3B illustrates the relationship between distinctness of a CV partition collection and the performance of Elastic Net on that partition collection for one gene. Supplementary Fig. 3C shows the distribution of the Spearman correlation values calculated between performance and distinctness – exemplified in Supplementary Fig. 3B – for all 100 genes.

SUPPLEMENTARY FIGURES:

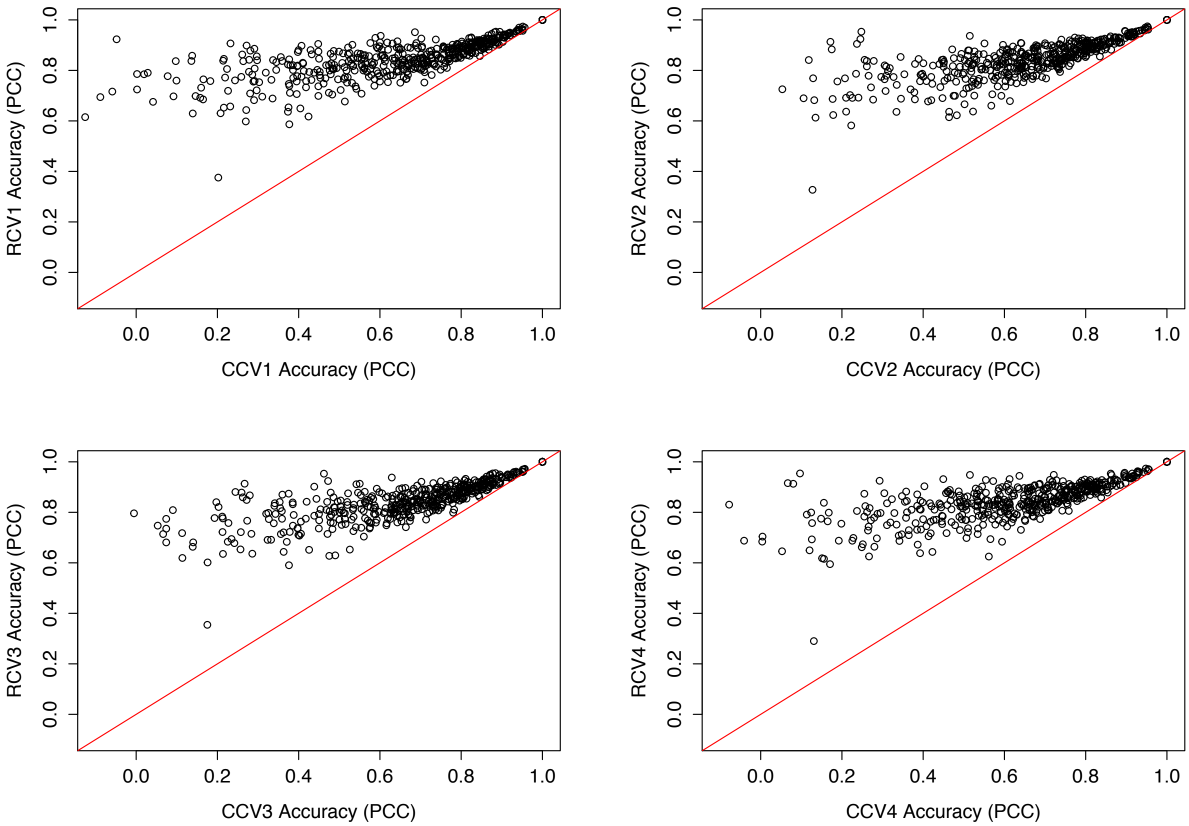

Supplementary Figure S1. Scatter plot showing the performance of LARS on each of the 500 genes using one of the 6-fold RCV partition collections (RCV1-RCV4) or one of the 6-fold CCV partition collections (CCV1-CCV4).

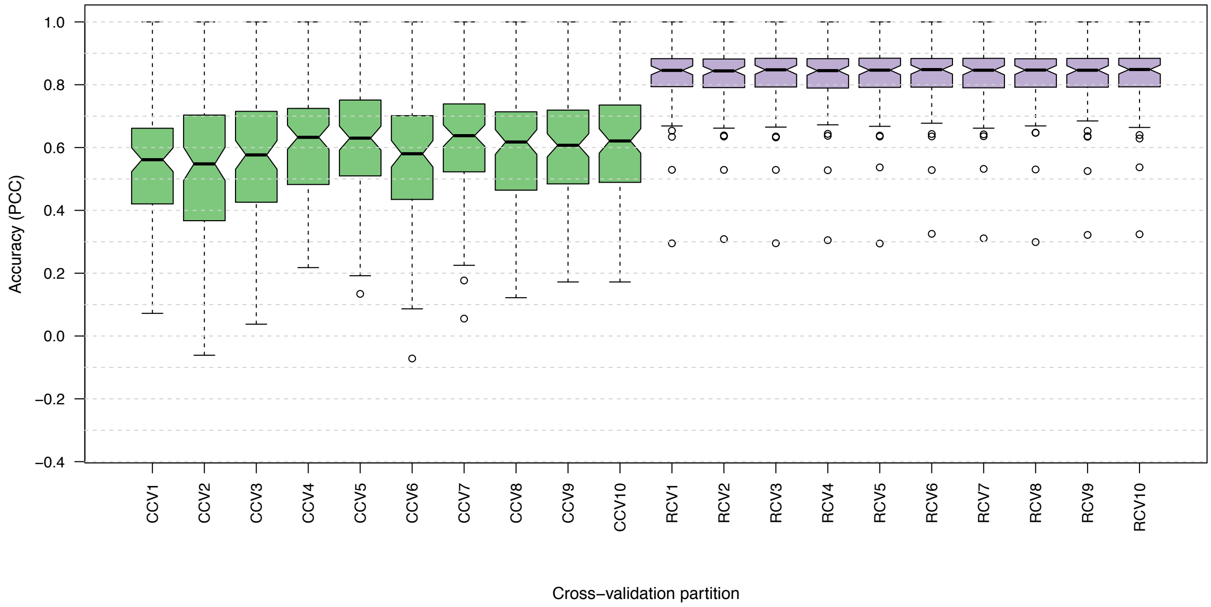

Supplementary Figure S2. Cross-validation performance of the Elastic Net regression model for gene expression prediction on the biased dataset. Distribution of the Pearson correlation coefficient between predictions (using Elastic Net) and real gene expression values over all test conditions. The test conditions are selected such that the number of samples from each cancer type is proportional to the number of samples of that cancer type in PanCan12. Each box plot shows the correlations calculated for 100 different genes. The ten boxplots labeled as CCV or RCV represent ten different 6-fold CV partition collections created using clustering-based or random partitions respectively. A higher Pearson correlation indicates higher accuracy.

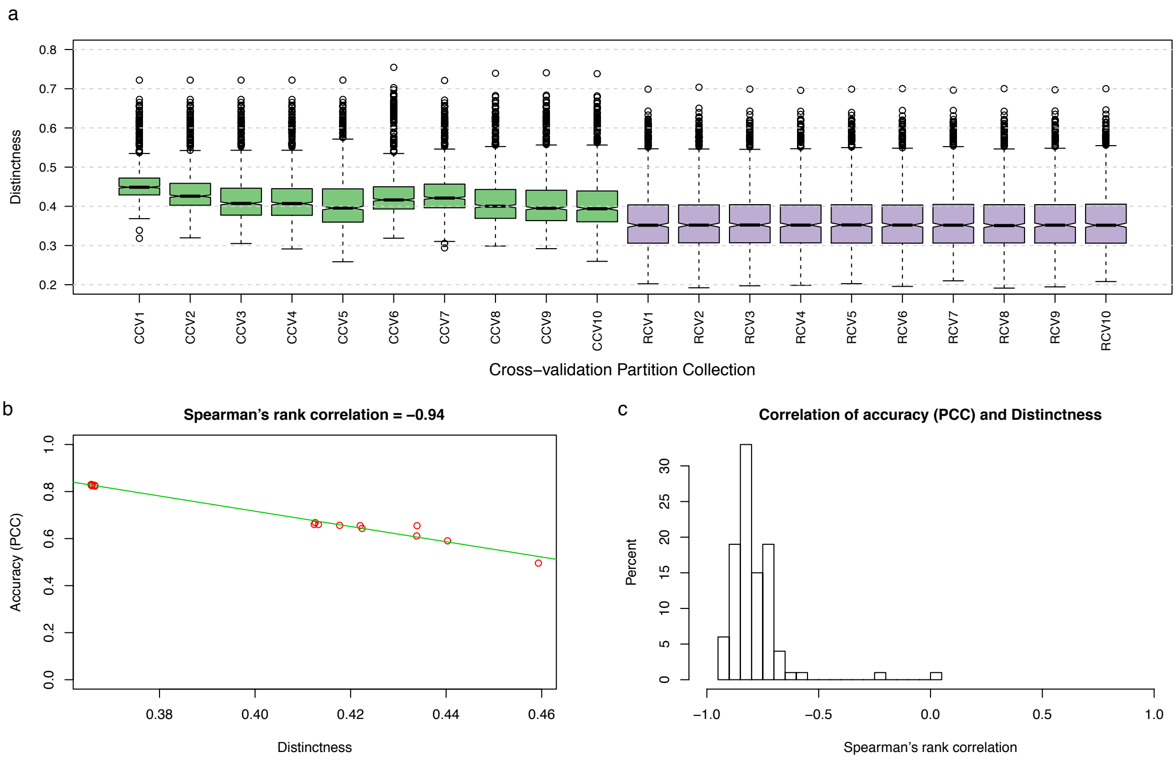

Supplementary Figure S3. Distinctness score of test conditions in clustered vs. random cross-validation schemes using the biased dataset. a) The distribution of distinctness score under various cross-validation schemes. Each boxplot represents distinctness scores of 864 conditions corresponding to a CV partition collection. b) The relationship between distinctness and performance of expression prediction for one gene. The plot shows the PCC values against the values of the distinctness scores for all 20 CV partition collections. Spearman's rank correlation between distinctness scores and PCC scores is equal to -0.94 for this gene. c) Histogram of the Spearman's rank correlation values between the distinctness and the prediction accuracy for 100 genes. Each correlation value is calculated as shown in Supplementary Figure S3b.

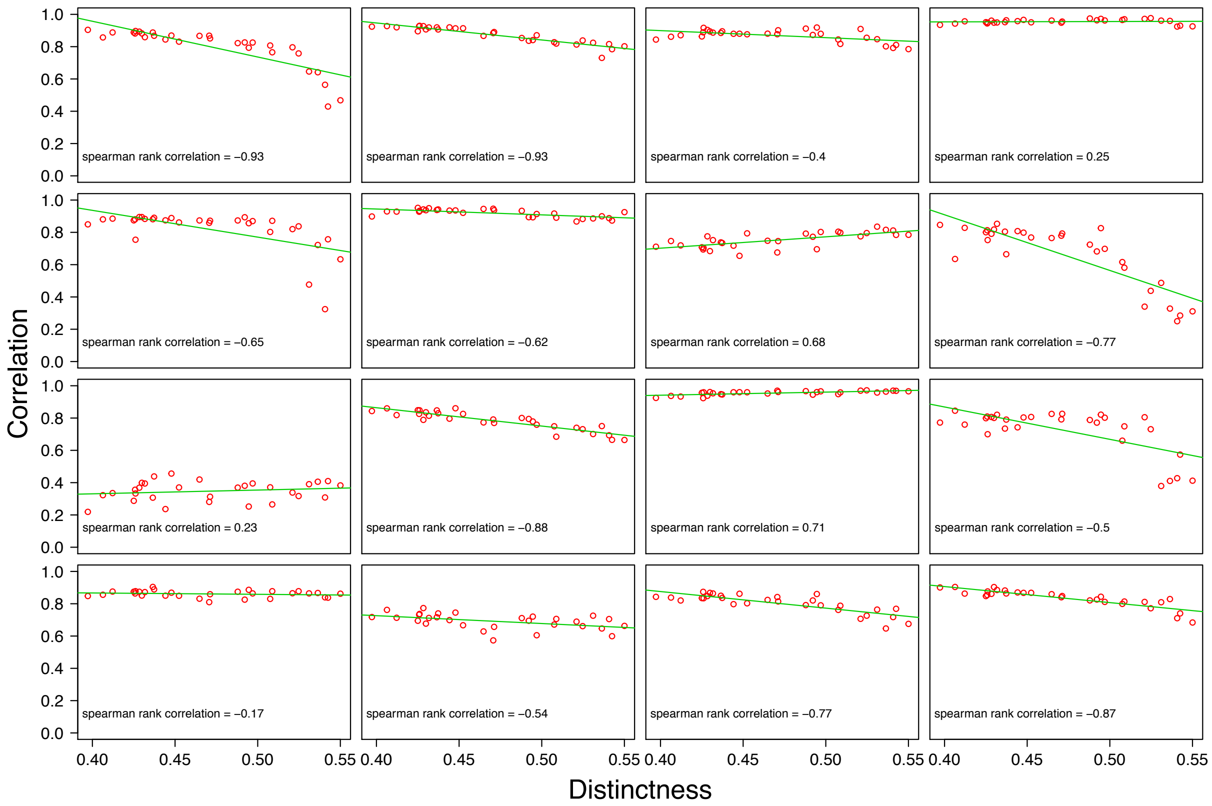

Supplementary Figure S4. Distinctness of test fold and their corresponding LARS performance (Pearson correlation) for 16 randomly chosen genes. Each plot shows 30 points, each corresponding to average distinctness score and LARS correlation on one test fold. Spearman rank correlation is shown for each gene.

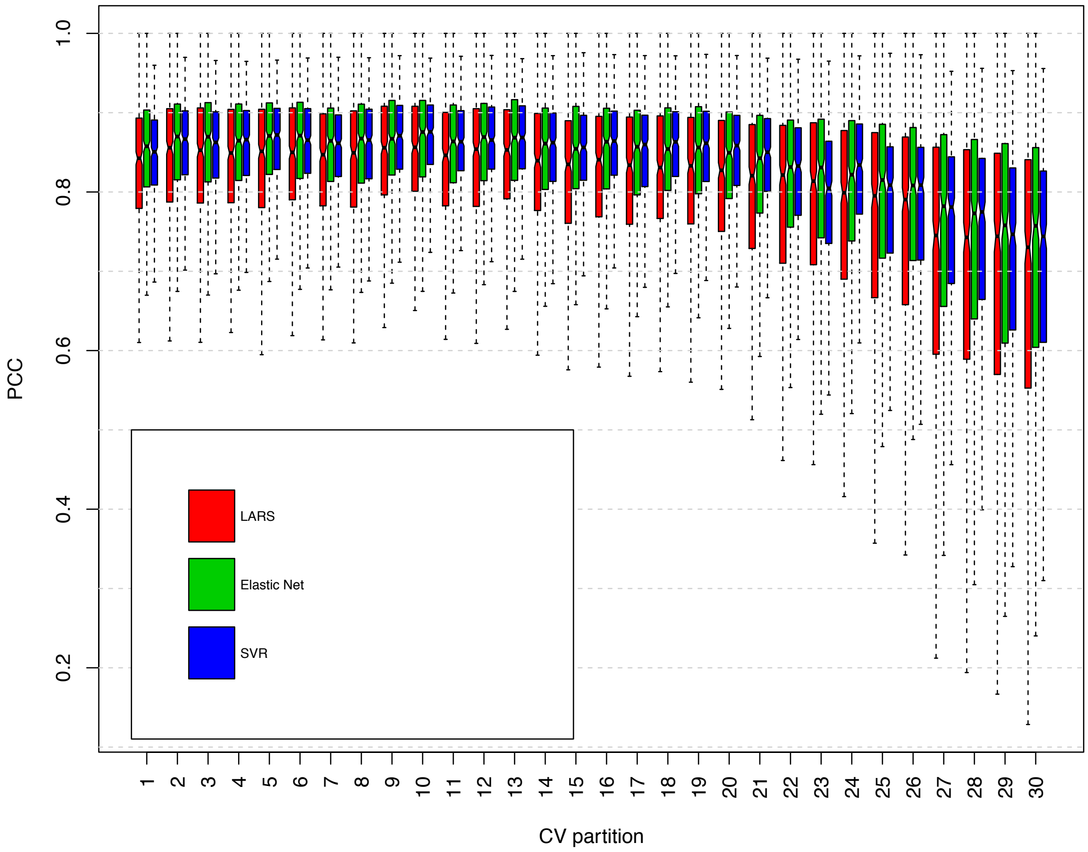

Supplementary Figure S5. The distribution of the test set prediction accuracies for 500 genes using three regression methods (LARS, Elastic Net and SVR) for the CV partitions obtained using simulated annealing. The prediction accuracy is measured using the PCC of model-predicted expression values and real expression values.

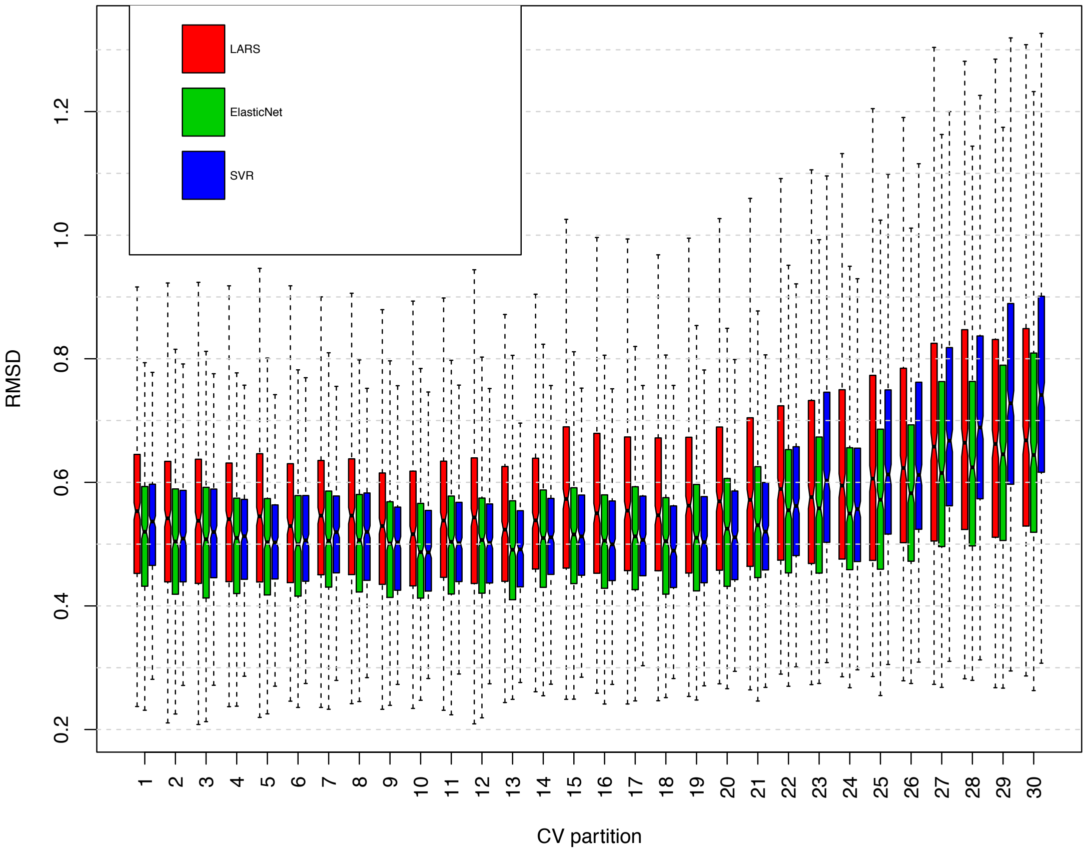

Supplementary Figure S6. The distribution of the test set prediction accuracies for 500 genes using three regression methods (LARS, Elastic Net and SVR) for the CV partitions obtained using simulated annealing. The prediction accuracy is measured using the RMSD of model-predicted expression values and real expression values.
